# Upregulation of Biomarker Limd1 Was Correlated with Immune Infiltration in Doxorubicin-Related Cardiotoxicity

**DOI:** 10.1155/2023/8347759

**Published:** 2023-03-23

**Authors:** Rui Zhang, Chunshu Hao, Zhenjun Ji, Yangyang Qu, Wenjie Zuo, Mingming Yang, Pengfei Zuo, Abdlay Carvalho, Genshan Ma, Yongjun Li

**Affiliations:** Department of Cardiology, Zhongda Hospital, School of Medicine, Southeast University, 87 Hunan Road, Nanjing, Jiangsu 210000, China

## Abstract

Doxorubicin is one of the most common antitumor drugs. However, cardiotoxicity's side effect limits its clinical applicability. In the present study, Gene Expression Omnibus (GEO) datasets were applied to reanalyze differentially expressed genes (DEGs) and construct weighted correlation network analysis (WGCNA) modules of doxorubicin-induced cardiotoxicity in wild-type mice. Several other bioinformatics analyses were performed to pick out the hub gene, and then the correlation between the hub gene and immune infiltration was evaluated. In total, 120 DEGs were discovered in a mouse model of doxorubicin-induced cardiotoxicity, and PF-04217903, propranolol, azithromycin, etc. were found to be potential drugs against this pathological condition. Among all the DEGs, 14 were further screened out by WGCNA modules, of which Limd1 was upregulated and finally regarded as the hub gene after being validated in other GEO datasets. Limd1 was upregulated in the peripheral blood mononuclear cell (PBMC) of the rat model, and the area under curve (AUC) of the receiver operating characteristic curve (ROC) in diagnosing cardiotoxicity was 0.847. The GSEA and PPI networks revealed a potential immunocyte regulatory role of Limd1 in cardiotoxicity. The proportion of “dendritic cells activated” in the heart was significantly elevated, while “macrophage M1” and “monocytes” declined after in vivo doxorubicin application. Finally, Limd1 expression was significantly positively correlated with “dendritic cells activation' and negatively correlated with “monocytes” and “macrophages M1'. In summary, our results suggested that limd1 is a valuable biomarker and a potential inflammation regulator in doxorubicin-induced cardiotoxicity.

## 1. Introduction

Thanks to the advancements in science and technology, numerous treatment schemes have been developed, which have gradually extended the survival times of tumor patients. Chemotherapy drugs including doxorubicin played a vital role in achieving this trend. Doxorubicin, also known as adriamycin, is a member of the anthracycline antibiotics with decades of history [1]. In the antitumor field, doxorubicin is widely applied in clinical settings for the treatment of various types of tumors, from soft tissue and bone sarcomas, cancers of the breast, ovary, bladder, thyroid and lung, acute lymphoblastic leukemia, acute myeloblastic leukemia, Hodgkin lymphoma, etc. [2].

In addition to its unique therapeutic function, this classic agent can lead to a series of side effects in the course of treatment including cognitive impairment [3], hepatotoxicity [4], bone marrow toxicity [5], nephrotoxicity [6], and especially cardiotoxicity [7]. The undesirable effect of cardiotoxicity is especially prominent during the whole treatment period and is usually characterized by irreversible degenerative dilated cardiomyopathy (DCM) and consequently resulting in congestive heart failure (CHF), thus greatly worsening the long-term outcomes of patients [8]. Several mechanisms have been proposed to account for this phenomenon, including the generation of reactive oxygen species (ROS), activation of apoptosis, calcium dysfunction, as well as induction of endothelin-1 (ET-1) and topoisomerase-II [9]. However, understanding this pathological process is still in its infancy, and the specific mechanism remains to be elucidated.

The development of bioinformatics technology has brought great opportunities for fully understanding doxorubicin-induced cardiotoxicity. In the present study, we reanalyzed two datasets GSE23598 and GSE59672 from the GEO database to clarify the differentially expressed genes (DEGs) and the coexpression modules in the doxorubicin-mouse model. Several genes screened out from DEGs and modules were validated in other in vivo model or in vitro model datasets to screen a key gene and evaluate its diagnostic value. According to the analysis results, the hub gene was regarded as inflammation-related, and finally, its correlation with immune infiltration in diseased hearts was calculated.

## 2. Materials and Methods

### 2.1. Bioinformatic Datasets

By searching the Gene Expression Omnibus (GEO, https://www.ncbi.nlm.nih.gov/geo/) database, five in vivo experiment datasets GSE23598, GSE59672, GSE81448, GSE97642, and GSE37260 were obtained. In GSE23598, 4 male wild-type (WT) mice aged 6-8 weeks were randomly assigned to the doxorubicin group (*n* = 2) and control group (*n* = 2) following injection with a single dose of doxorubicin (15 mg/kg, i.p.) or an equal volume of normal saline, respectively. Animals were sacrificed on the 4th day after injection, and hearts were collected for further detection of gene expression profiling by microarray with platform GPL1261. In GSE59672, six male WT mice aged 10-12 weeks were randomly assigned to 2 groups (*n* = 3 in each group) and received similar treatment as the mice from the GSE23598 database, and were sacrificed on the 5th day. The gene expression profiling was performed with the same microarray platform GPL1261. In both GSE81448 and GSE97642, male mice aged 8-10 weeks were injected intraperitoneally with 15 mg/kg doxorubicin (*n* = 5 in each dataset) or an equal amount of PBS (*n* = 5 in each dataset). Mice were euthanized 20 hours after injection, and the expression profiles of the diseased heart were detected both by microarray with platform GPL6887. In GSE37260, rats were treated with doxorubicin (*n* = 9) or saline (*n* = 8) for 48 hours, and the peripheral blood mononuclear cell (PBMC) was used for detecting transcriptome expression with the bead chip platform GPL6101.

Furthermore, three in vitro experiment datasets GSE42177, GSE154101, and GSE154118 were enrolled. In GSE42177, cardiac myocytes (CMs) were treated with doxorubicin for 0 hr (control group, *n* = 3) and 8 hrs (doxorubicin group, *n* = 3). The gene expression profiling was detected by microarray on platform GPL6247. In GSE154101, fibroblasts (FBs) of human pulmonary arterial adventitial were isolated to treat with vehicle (control group, *n* = 2) and doxorubicin (doxorubicin, *n* = 2). In GSE154118, smooth muscle cells (SMCs) of the human pulmonary artery were treated with vehicle (control group, *n* = 2) and doxorubicin (doxorubicin, *n* = 2). These two datasets detected the expression profile by high-throughput sequencing with GPL18573.

### 2.2. Identification of Differentially Expressed Genes

Expression profiles of GSE23598, GSE59672, GSE81448, GSE97642, GSE37260, and GSE42177 were downloaded from the GEO database through the “GEOquery” package of R software. The probes corresponding to multiple targets were removed. When multiple probes corresponded to the same molecule, only the probe with the largest signal value was retained. After filtering the data, the combat function of the “SVA” package was used to remove the interbatch difference among GSE23598 and GSE59672 datasets, as well as GSE81448 and GSE97642. Then the standardization situation was viewed through the box chart, and the clustering of samples was checked with the PCA diagram and UMAP diagram. The gene expression profile of GSE37260 was performed with normalized quantiles. Finally, the “limma” package was used to analyze the differentially expressed genes (DEGs) between control and doxorubicin groups in datasets performed with microarray. Besides, DEGs of datasets GSE154101 and GSE154118 were analyzed in the well-known web tool NetworkAnalyst (https://www.networkanalyst.ca/) [10] with the DESeq2 method according to the instruction.

The screening criteria for DEGs are as follows: log_2_ fold change (FC) >1 or < -1, and adjusted *p* value (adj. p. val) <0.05. If log_2_ FC>1, the expression of DEGs was deemed as upregulated, while < -1 was downregulated.

### 2.3. Weighted Gene Coexpression Network Analysis

Weighted gene coexpression network analysis (WGCNA) is a systematic biological method for counting the correlation patterns among genes across microarray profiles [11]. The online tool EHBIO platform (http://www.ehbio.com/Cloud_Platform/front/#/) was used to perform WGCNA on the combined dataset (GSE23598 and GSE59672). Modules most related to traits of the WGCNA were enrolled for the following study after the screening.

### 2.4. Search of Upstream miRNAs

The expression of genes is usually regulated by miRNAs at the posttranscriptional level [12]. Therefore, the upstream miRNAs of the DEGs in GSE23598 and GSE59672 were searched from the miRNET website (https://www.mirnet.ca/) [13]. The miRNA-mRNA networks were subsequently constructed from this web tool.

### 2.5. Protein-Protein Interaction Network Construction

The “Search Tool for Retrieval of Interacting Genes/Proteins” database (STRING, https://string-db.org/) provides abundant information on protein-protein interaction (PPI) [14], and those DEGs encoded proteins were incorporated into the PPI network with STRING. Then, network visualization was performed using Cytoscape software (https://cytoscape.org/), an open-source software platform [15].

### 2.6. Potential Therapeutic Drugs Prediction

The Connectivity Map (cMap) database can provide information about the effects of small molecules, genes, or diseases on gene expression signatures [16], and it was applied to search for potential drugs against doxorubicin-induced cardiotoxicity. The online application CLUE (https://clue.io) [17] provides a convenient entrance for the use of the cMAP database. The candidate DEGs were divided into upregulated group and downregulated group and imported into CLUE to calculate the score of all factors including small molecules. When the score is negative, it is considered that small molecules have antagonistic effects. Conversely, when the score is positive, small molecules will be considered to have the effect of exacerbating cardiotoxicity.

### 2.7. Functional Enrichment Analysis

Gene ontology (GO) analysis mainly includes three aspects: molecular function (MF), biological process (BP), and cellular component (CC) [18]. The Kyoto Encyclopedia of Genes and Genomes (KEGG) database provides biological signaling pathway information enriched by a group of genes [19]. The DAVID database (https://david.ncifcrf.gov/) [20], a comprehensive set of functional annotation tools for understanding the biological meaning of genes, was freely accessed and applied to explore the GO and KEGG results.

### 2.8. Identifying Biomarker and Hub Gene in Disease Progression

DEGs of the mouse model and the selected WGCNA modules were intersected. The overlapping genes were validated in the merged datasets (GSE81448 and GSE97642), and the three in vitro datasets (GSE42177, GSE154101, and GSE154118) and the rat PBMC dataset (GSE37260) in turn to pick out the hub gene. Gene Set Enrichment Analysis (GSEA) is a knowledge-based approach for analyzing groups of genes that share common biological functions, chromosomal locations, or regulations [21]. The GSEA was analyzed according to groups divided by hub gene with the Sangerbox tools (http://sangerbox.com/Tool), a free online platform. Online database GeneMANIA (http://genemania.org/), a user-friendly web tool for generating hypotheses about gene function, analyzing gene lists, and prioritizing genes for functional assays [22], was used to evaluate the biological function of the hub gene by searching its related genes. GraphPad Prism 8.0 was used to display the expression distribution and perform receiver operating characteristic (ROC) statistical analysis of the hub gene to evaluate its diagnostic efficacy.

### 2.9. Immune Infiltration Analysis

CIBERSORTx (https://cibersortx.stanford.edu/) [23] is an analytical tool that provides an estimation of the abundances of member cell types in a mixed cell population using a gene expression matrix. The combination of GSE23598 and GSE59672 was applied for analysis in the CIBERSORTx web tool to evaluate the percentages of 22 kinds of infiltrating immunocytes. Statistical analysis was done using Pearson and Wilcoxon test methods. *p* < 0.05 was considered statistically significant. Finally, the correlation between hub gene expression level and scores of immune infiltration was calculated using the Pearson correlation coefficient in GraphPad Prism 8.0.

## 3. Results

### 3.1. Identification of DEGs between Control and Doxorubicin Mouse Model

**Figure 1:**
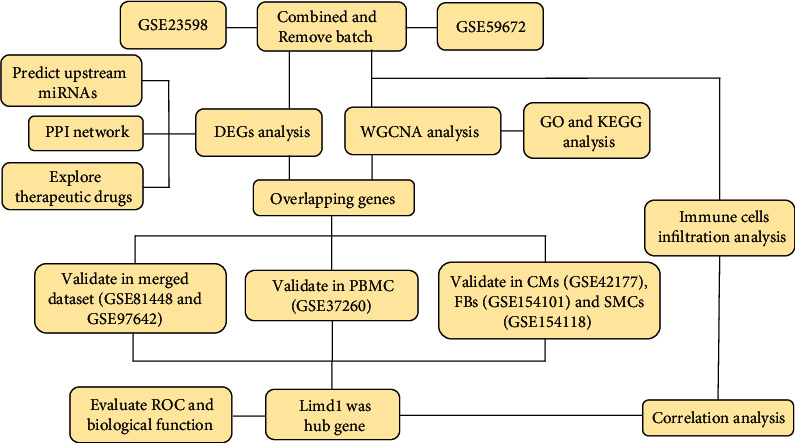
The workflow of this study. DEGs: differentially expressed genes; WGCNA: Weighted Gene Coexpression Network Analysis; miRNAs: microRNAs; PPI: protein-protein interaction; GO: gene ontology; KEGG: Kyoto Encyclopedia of Genes and Genome; PBMC: peripheral blood mononuclear cell; CMs: cardiomyocytes; FBs: fibroblasts; SMCs: smooth muscle cells; ROC: receiver operating characteristic.

**Figure 2:**
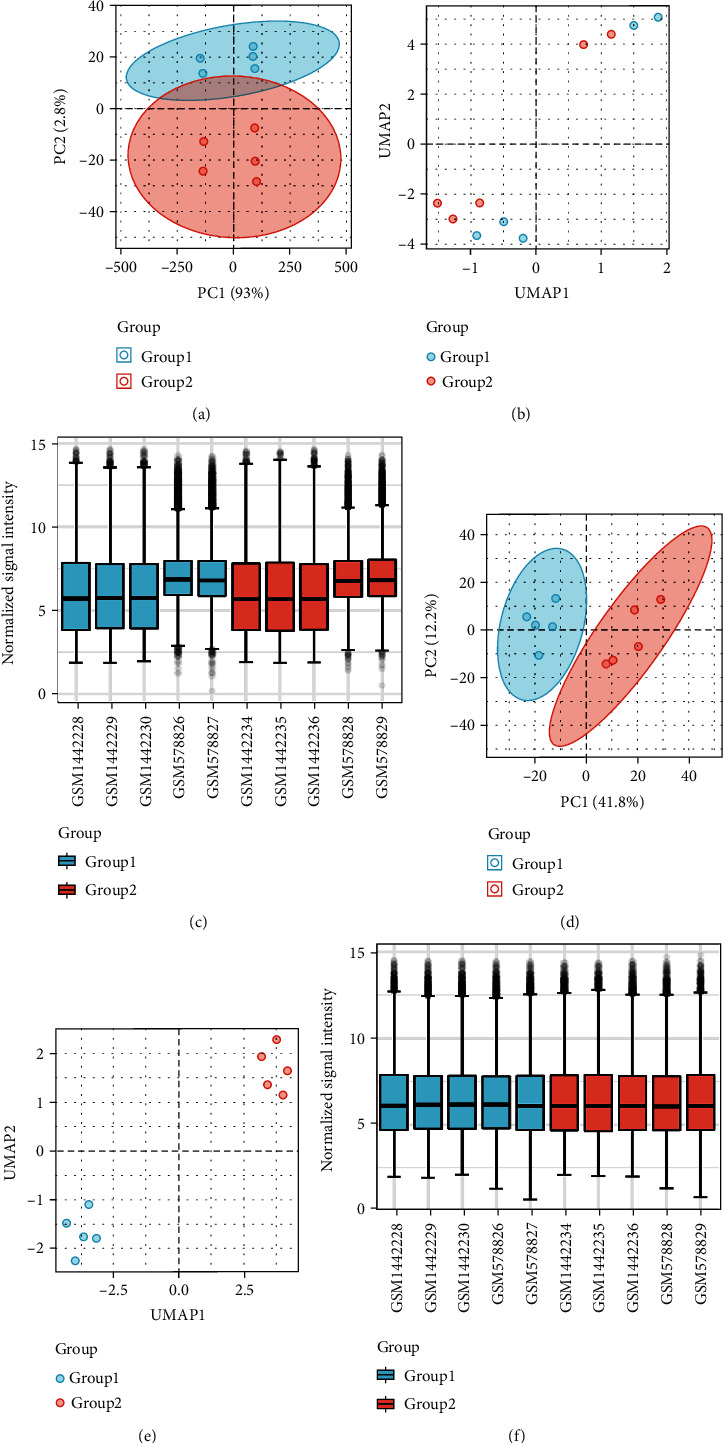
Remove batch between GSE23598 and GSE59672. (a–c) PCA diagram, UMAP diagram, and boxplot diagram of the data distribution before removing the batch. (d–f) PCA diagram, UMAP diagram, and boxplot diagram of the data distribution after removing the batch. Group 1 represents control group, while group 2 represents the doxorubicin-treated group.

**Figure 3:**
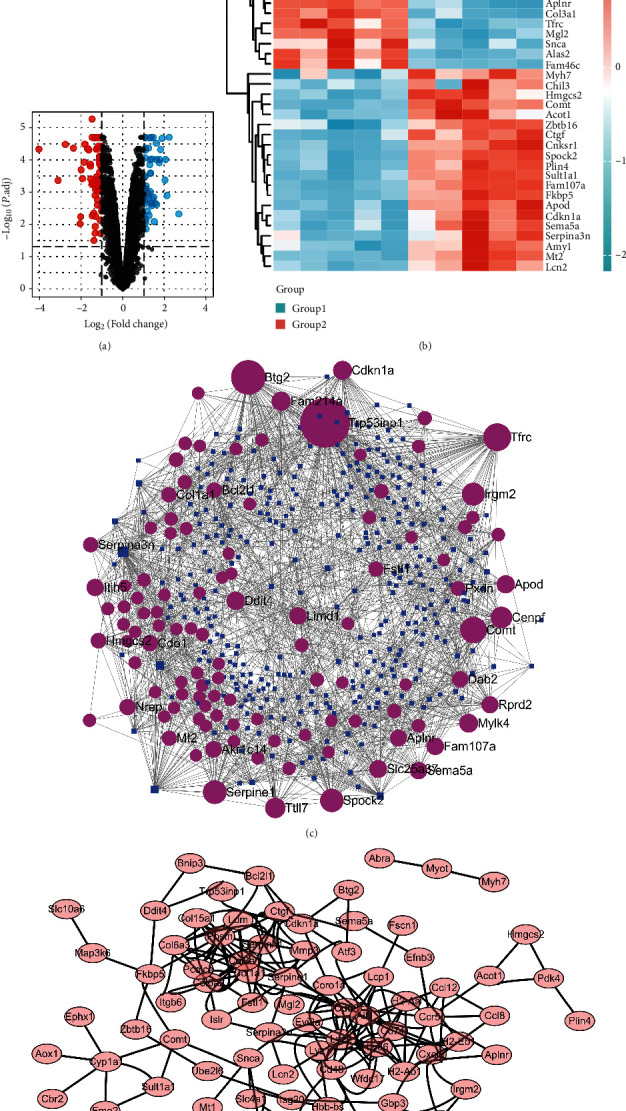
Construct miRNA-mRNA network and PPI network based on DEGs of the combined dataset. (a) The volcano plots indicated the DEGs. (b) The heatmap of the top 20 upregulated and downregulated DEGs. Group 1 represents the control group, while group 2 represents the doxorubicin-treated group. (c) The miRNA-mRNA network. (d) The PPI network.

The whole research process is summarized in Figure 1. In accordance with the study design, the batch differences between expression profiles in datasets GSE23598 and GSE59672 regarding wild-type mice were first removed, and the data quality before (Figures 2(a)–2(c)) and after (Figures 2(d)–2(f)) batch removal was assessed and displayed in form of PCA, UMAP, and boxplot. Totally, there were 20813 genes enrolled in the final expression matrix (Table 1). However, only 120 genes were considered to be differentially expressed between 5 control samples and 5 doxorubicin samples according to the criterion: |log2(FC)| > 1 and an adjusted *p* value <0.05 (Figure 3(a)). The expression level of the top 20 upregulated and downregulated genes is shown in the heatmap (Figure 3(b)). Among these 120 DEGs, 54 were upregulated and 66 were downregulated. The number of DEGs under different criteria is represented in Table 1, and detailed information on the 120 DEGs is listed in Supplementary Table 1.

### 3.2. Network Analysis of DEGs

MiRNAs are vital regulators of mRNA expression and will form a tight regulatory system with these upregulated or downregulated DEGs. Therefore, the upstream miRNAs were searched with an online tool, and 334 miRNAs were finally revealed to target 109 of the 120 DEGs to form a complex miRNA-mRNA network with 1773 edges (Figure 3(c)). As shown, miR-155-5p, mir-122-5p, and miR-1a-3p have the most downstream target DEGs. Besides, the mRNA-encoded proteins could potentially interact with each other. The PPI network provides information of multiple levels of information regarding the interaction; therefore, the STRING database was used to construct the PPI network, and Cytoscape software was used to visualize the 65 nodes and 205 edges contained in the PPI network (Figure 3(d)).

### 3.3. Predicting Potential Therapeutic Drugs

The cMAP database provides information about the action of a series of small molecules on the gene expression profiles of several cell lines. By matching the 54 upregulated and 66 downregulated genes to the database, several small molecules including PF-04217903, propranolol, and azithromycin (Table 2) were highlighted as most likely to fight against doxorubicin-induced cardiotoxicity.

### 3.4. Construction of Weighted Gene Coexpression Network and Identify Key Modules

**Figure 4:**
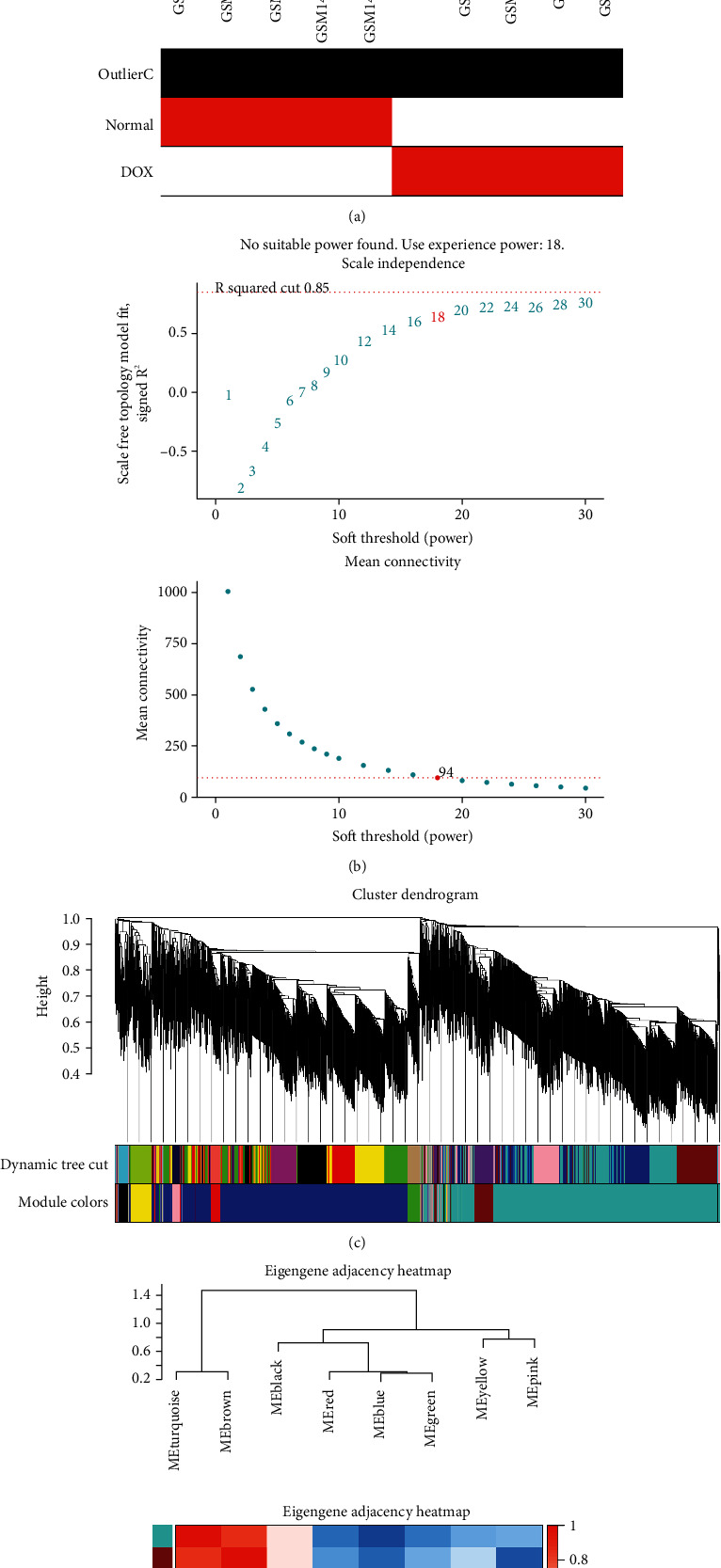
WGCNA indicated the coexpression modules. (a) Sample clustering to detect outliers. (b) Analysis of network topology for a set of soft-thresholding powers. (c) Clustering dendrograms of genes with dissimilarity based on the topological overlap and the assigned module colors. (d) The eigengene dendrogram and heatmap identify groups of correlated eigengenes.

WGCNA was performed to identify the gene set and construct its connection with phenotype. Hierarchical clustering analysis was performed, and there existed no outlier (Figure 4(a)). Network topology analysis was used to determine candidate power values for relative, balanced scale independence, and mean connectivity in the WGCNA, and finally, the soft-thresholding power was selected as 18 after comprehensive consideration (Figure 4(b)). Totally, there were 8 coexpression modules screened out. The modules containing the most genes were the turquoise and blue modules (Figure 4(c)). The eigengene network indicated that there is good discrimination between different modules (Figure 4(d)).

**Figure 5:**
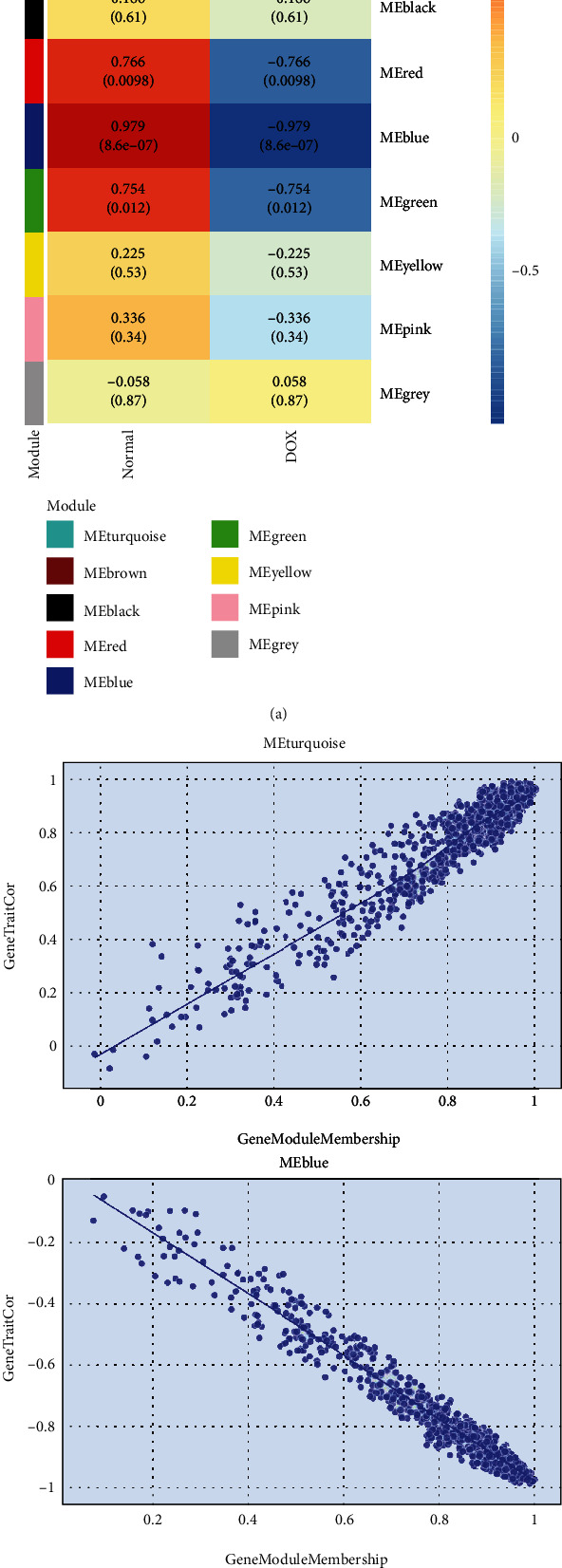
Evaluation of the correlation between modules and the phenotype. (a) Heatmap of the correlations between the module eigengenes and disease trait. (b) Scatterplot of gene significance (GS) vs. module membership (MM) in turquoise and blue.

A heatmap of module-trait correlations analysis showed that multiple modules were related to doxorubicin-induced cardiotoxicity, while MEturquoise had a maximum positive correlation coefficient and MEblue had a maximum negative correlation coefficient (Figure 5(a)). Finally, genes in the two modules were evaluated for their correlation with the trait (gene significance) and module (module membership), respectively, and displayed in a scatterplot (Figure 5(b)). There was a significant correlation between gene significance scores and module membership scores between the two modules.

### 3.5. Functional Enrichment Analysis of the Modules

**Figure 6:**
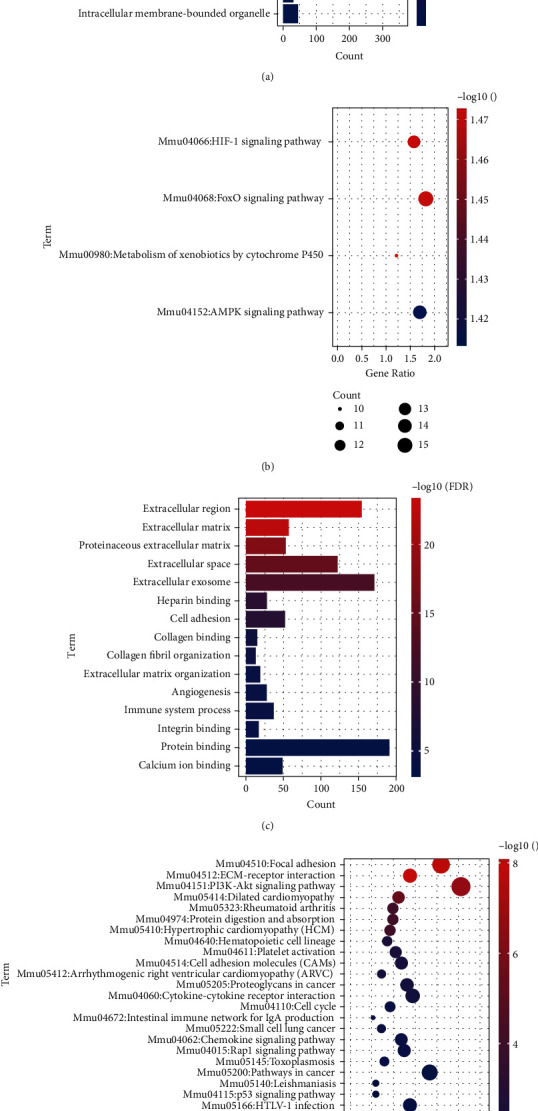
Biological characteristics of the genes in MEturquoise and MEblue. (a) Gene Ontology analysis of MEturquoise. (b) KEGG pathway enrichment of MEturquoise. (c) Gene Ontology analysis of MEblue. (d) KEGG pathway enrichment of MEblue.

To further understand the biological effects and signaling pathway enrichment of MEturquoise and MEblue, GO and KEGG analyses were performed. Genes in MEturquoise were enriched in “cytoplasm,” “nucleotide binding,” “cellular response to insulin stimulus,” etc. of the GO project (Figure 6(a)). “HIF-1 signaling pathway,” “FoxO signaling pathway,” etc. were key biological pathways involved by the MEturquoise (Figure 6(b)). In MEblue, genes were enriched in the “extracellular region,” “heparin-binding,” and “cell adhesion” (Figure 6(c)). Besides, KEGG analysis indicated that the biological pathway enriched in MEblue is different from that in MEturquoise, which included “focal adhesion,” “ECM-receptor interaction,” and “PI3K-Akt signaling pathway,” (Figure 6(d)).

### 3.6. Screen out Key Genes to Validate and Evaluate their Expression Distribution

**Figure 7:**
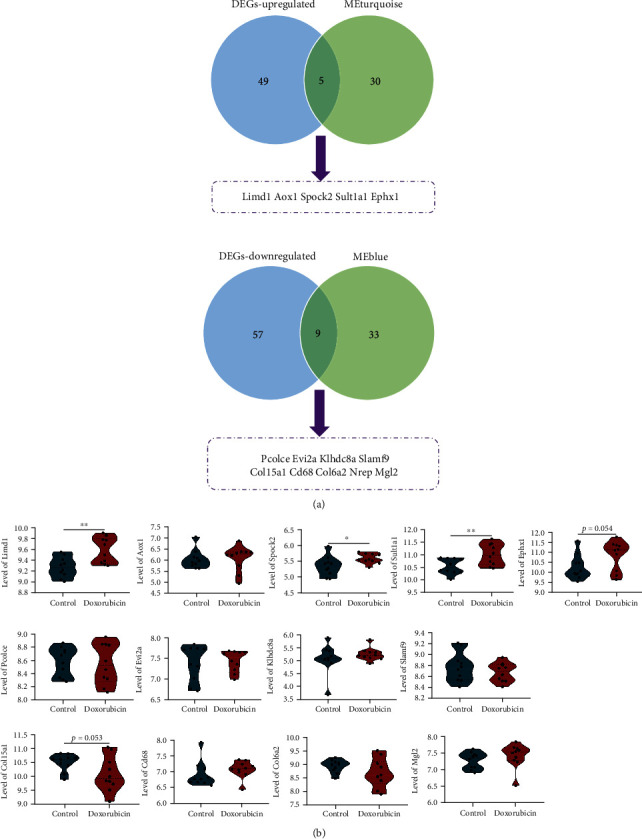
Validate the expression of key genes screened out by DEGs and EGCNA. (a) The overlapped genes between upregulated genes and selected genes in the MEturquoise, as well as downregulated genes and selected genes in the MEblue. (b) Expression of the 14 overlapping genes in the merged dataset of GSE41884 and GSE97642.

The genes located in the upper right region of the MEturquoise scatterplot and the lower right of the MEblue scatterplot were regarded as potential vital factors. Therefore, genes with gene significance and module membership both scored more than 0.95 and were selected to evaluate their differential expression. As shown in Figure 7(a), 35 genes were selected out from MEturquoise, of which 5 were significantly upregulated, and 9 of 42 genes selected out from MEblue were significantly downregulated in doxorubicin-induced cardiotoxicity (GSE23598 and GSE59672). To assess the role of these 14 hub genes that were key in the progression of cardiotoxicity, we first assessed their expression in the merged dataset of GSE81448 and GSE97642. The PCA, UMAP, and boxplot analyses of these two datasets before and after the combination was displayed in Supplementary Figure 1. Finally, several genes including Limd1 were significantly dysregulated (*p* < 0.05), but the log_2_ FC was all less than 1 (Figure 7(b)).

The cardiac tissue mainly consisted of CMs, FBs, SMCs, and immune cells. In order to explore the main effector cell population leading to changes in these 14 key genes, we reanalyzed the datasets (GSE42177, GSE154101, and GSE154118) that evaluate the expression matrix of doxorubicin-treated CMs, FBs, and SMCs, respectively. The analysis results of DEGs were displayed in Supplementary Figure 2 in the form of a volcano plot, and detailed results of the 14 genes including log_2_ FC and adj.*p* value were summarized in Table 3. According to Table 3, several genes were upregulated or downregulated, most likely contributed by immune cells. For example, Limd1 was significantly downregulated in CMs induced by doxorubicin but not significantly changed in CFs and SMCs. However, its expression was significantly upregulated in the organizational whole composed of the above elements, which indicates that inflammatory cells may have led to this change trend.

### 3.7. Limd1 Was a Potential Valuable Circulating Biomarker in the Cardiotoxicity

**Figure 8:**
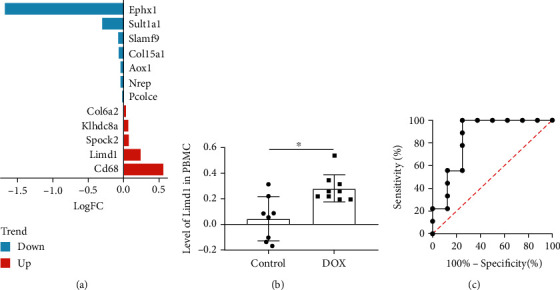
Validate the expression and evaluate the biomarker role of Limd1. (a) The change characteristics of gene expression of the overlapping genes in PBMC of doxorubicin-cardiotoxicity model. (b) The relative level of Limd1 in PBMC. (c) ROC analysis of Limd1.

Due to that the above key genes may be dysregulated in the immune cells, we further evaluated their expression patterns of them in the circulating PBMC of the doxorubicin-related cardiotoxicity model (GSE37260). Among them, the expression of 12 genes was detected, while Evi2a and Mgl2 were nonexistent (Figure 8(a)). Further, Limd1, Spock2, Pcolce, Slamf9, Col15a1, and Nrep were consistent with those in the heart of the mouse model in terms of differential expression trend, while only the dysregulation of Limd1 was significant (adj.*p*.val < 0.05). Limd1 was upregulated in the PBMC of the rat treated with doxorubicin (Figure 8(b)), and the area under the ROC curve for Limd1 in diagnosing the cardiotoxicity was 0.8472 (Figure 8(c)), suggesting that Limd1 is a valuable circulating biomarker to judge this diseased state.

### 3.8. GSEA of Limd1-Associated Gene Set

**Figure 9:**
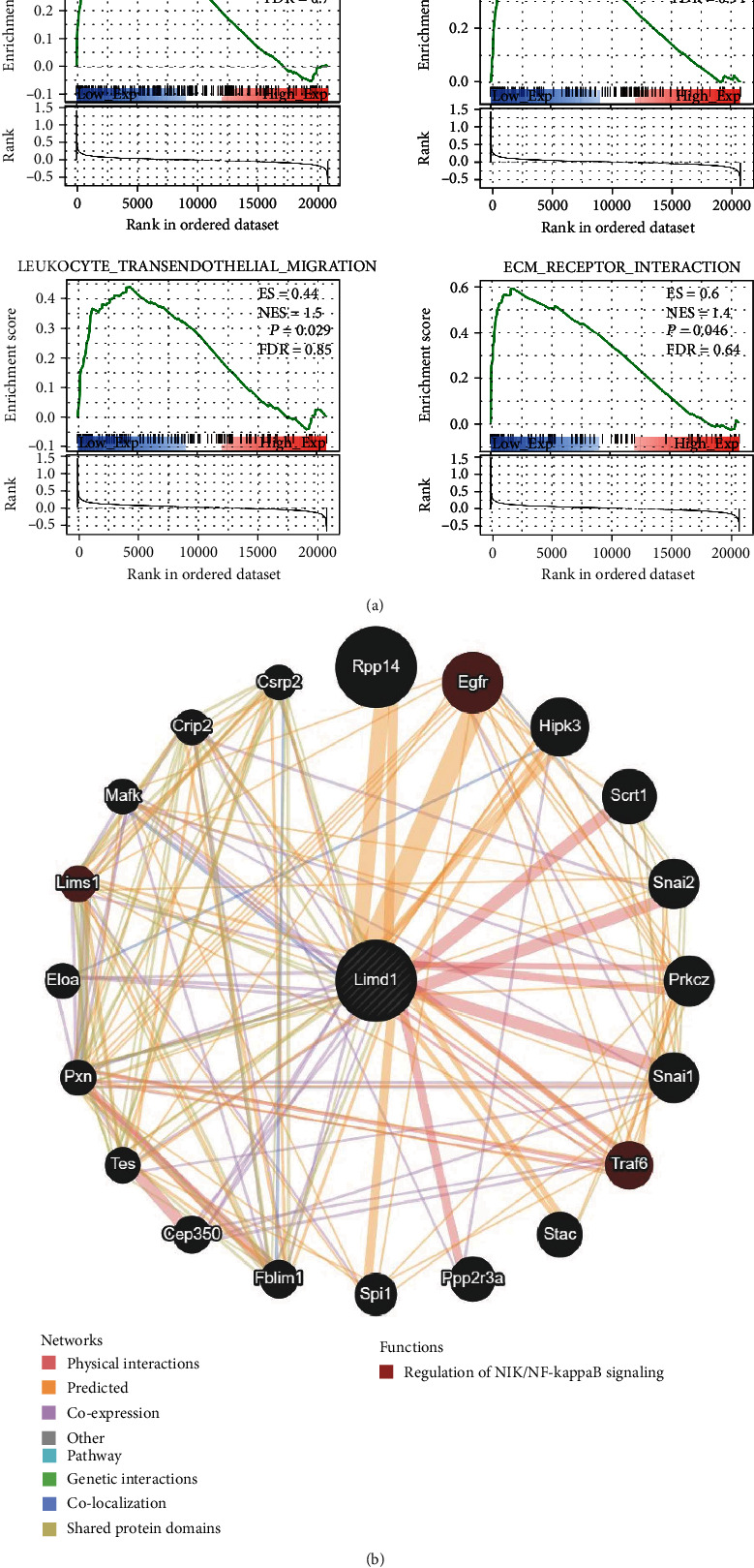
Explore the biological function of Limd1. (a) GSEA was used to analyze the signaling pathways enrichment in different groups according to Limd1. (b) Network of protein interaction with Limd1.

In order to explicit the biological characteristic involved by Limd1, we ranked genes from the 10 normal and diseased heart samples in the merged dataset by their relative Limd1 expression in the top 50% vs. the bottom 50% for GSEA analysis based on the KEGG database. There were dozens of categories enriched (*p* < 0.05 but FDR > 0.25), and several are functionally closely related to inflammatory cells including the chemokine signaling pathway, cell adhesion molecules pathway, and leukocyte transendothelial migration signaling (Figure 9(a)). These pointed out that the key gene Limd1 was a possible regulator in controlling immunocyte infiltrating tissue.

To further explore the potential biological function of Limd1, its interaction protein network was constructed, and the result showed that Limd1 may participate in inflammatory regulation through the NF-kappaB signaling pathway (Figure 9(b)).

### 3.9. Assessment of Immune Infiltration and the Correlation with Limd1 Expression

**Figure 10:**
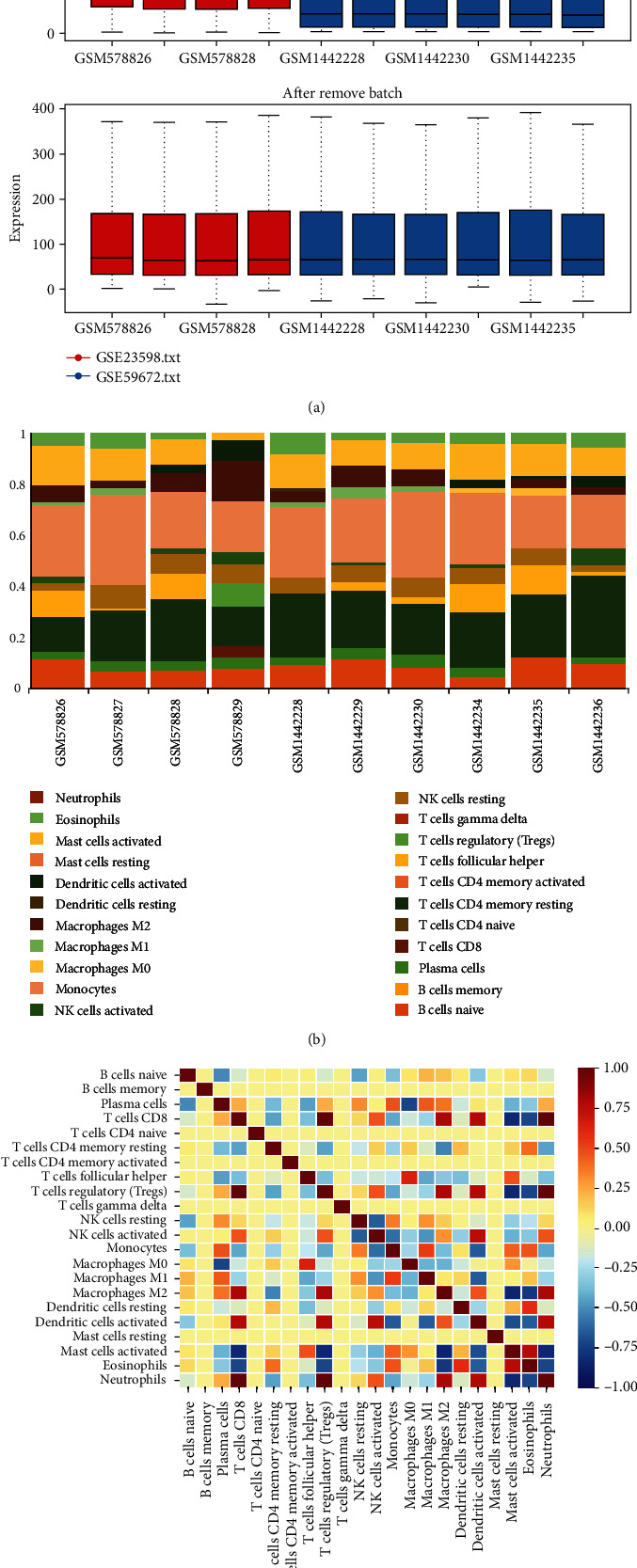
Estimate the immune infiltration of the heart. (a) Remove batch between original unlogged expression profile. (b) Barplot showed the composition of immune cells. (c) Heatmap showed the correlation among immune cells each other.

The primitive unlog transform microarray matrix regarding two mouse models was merged and removed batch for the subsequent immune infiltration assessment (Figure 10(a)). After being calculated in CIBERSORTx, the composition of immunocytes in the heart tissues of the doxorubicin-induced mouse model was indicated intuitively (Figure 10(b)). The heatmap displayed the correlation between one immunocyte with another (Figure 10(c)), and the results indicated that there exists a positive correlation between “activated dendritic cells” with “regulatory T cells (Tregs)” and “macrophages M2”, and a negative correlation between “activated dendritic cells” with “macrophages M1.”

**Figure 11:**
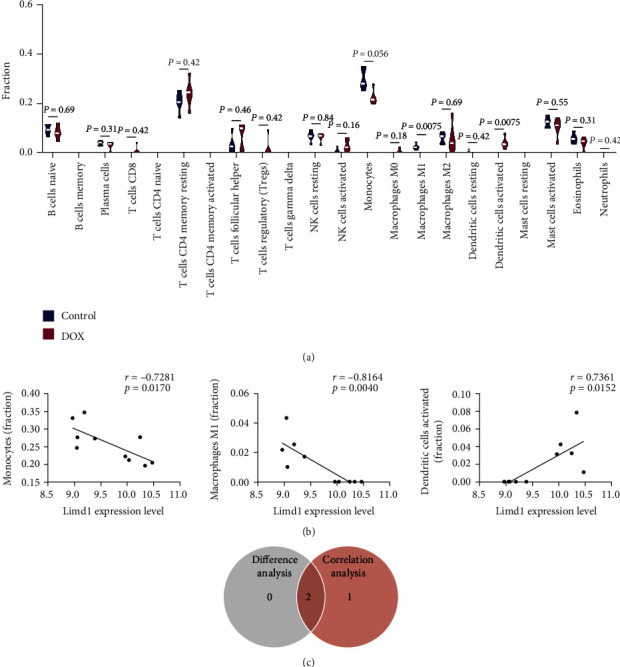
Evaluate the infiltration of immune cells and their correlation with Limd by Wilcoxon and Pearson correlation analysis. (a) The difference of immune infiltration between control and toxicity hearts. (b) The correlation between Limd1 expression with immune infiltration of monocytes, M1 macrophages, and activated dendritic cells. (c) Count the immunocytes with differential expression and correlate with Limd1, and intersect them.

The percentage data of each immunocyte type between the control and the doxorubicin groups were compared with the Wilcoxon test. As shown in the violin plot, the proportion of “dendritic cells activated” was elevated (*p* = 0.0075), while “macrophage M1' declined (*p* = 0.0075) in the doxorubicin group compared with the control group; the others showed no significance (Figure 11(a)). The correlation between Limd1 expression and the fraction of immunocytes was evaluated. Totally, 3 kinds of immunocytes were found to correlate with the Limd1 level. Among them, “activated dendritic cells” and “macrophage M1” were positively and negatively correlated with Limd1 level, respectively (Figure 11(b)). In addition, although “monocytes” did not change significantly (*p* = 0.056) under doxorubicin, it was negatively correlated with Limd1 level (Figures 11(b) and 11(c)).

## 4. Discussion

As a widely clinically used drug, doxorubicin has a very powerful antitumor function. However, a growing number of studies support the conception that doxorubicin plays a double-edged sword role during its application. Indeed, the action of doxorubicin on nontargeted tissues may result in cardiotoxicity and eventually worsen to congestive heart failure and death, which will disrupt cancer treatment by needing to control medical dosages of doxorubicin, thus deteriorating the quality of life of patients [24]. Therefore, exploring new strategies against the cardiotoxic effect of doxorubicin would benefit cancer patients to a certain extent.

In this study, we selected two datasets GSE23598 and GSE59672 from the GEO database as the subjects. These two datasets were based on a doxorubicin-induced acute heart injury mouse model performed through intraperitoneal administration of doxorubicin in a dose of 15 mg/kg for 4 or 5 days [25, 26]. After combining and reanalyzing the datasets, we screened out a total of 120 DEGs between the normal myocardial tissue and the toxic myocardial tissue, of which parts were confirmed by previous reports in terms of the expression trend, such as Ctgf [27], Aox1 [28], Alas2 [29], and Ly86 [30]. However, Btg2 was upregulated after doxorubicin treatment in our study, while a previous study found it to be downregulated [31]. On the whole, the differentially expressed genes obtained based on omics were relatively reliable. Furthermore, Cxcl9 was discovered to be downregulated in the heart tissue of the mouse model. Its protein level in plasma was evaluated in breast cancer patients who received doxorubicin, and it was significantly lower in the abnormal decline of the left ventricular ejection fraction (LVEF) group compared with the normal LVEF group [32], indicating that these DEGs are potential circulating biomarkers for clinical application of doxorubicin-related cardiotoxicity in the future. Besides, based on these DEGs, the potential therapeutic drugs against the cardiotoxicity were predicted, and a series of molecules including propranolol were selected. Interestingly, propranolol was reported to have a significant beneficial effect on cardiac injury induced by doxorubicin [33]. Therefore, it is worth expecting whether the other drugs, especially PF-04217903, could relieve this toxicity in the myocardium.

As is well known, miRNAs usually negatively participate in regulating the protein expression of genes through degradation or translational inhibition. We therefore further predicted the upstream miRNAs that target all the 120 DEGs and finally constructed the network containing 334 miRNAs and 109 DEGs targeted by them. In this whole network, miR-155-5p, miR-122-5p, miR-1a-3p, etc. were most closely related to these DEGs. As revealed in the clinical research, circulating miR-122-5p was increased after doxorubicin application in breast cancer patients [34]. In the mouse model, the circulating level of miR-122-5p was higher in cardiotoxicity than in the nontoxicity group after doxorubicin was applied. However, the circulating miR-122-5p was lower in the noncardiotoxicity model compared with the saline-treated model [35]. Besides, the level of circulating miR-122-5p before treatment in patients with myocardial injury after treatment was significantly higher than that in patients without myocardial injury after treatment and could predict adverse cardiac reactions to doxorubicin [34]. Therefore, whether the other miRNAs especially miR-155-5p and miR-1a-3p have clinical application potential is worth exploring.

Genes are usually synergistically involved in disease phenotypes. Accordingly, we extracted the coexpression modules from the whole gene matrix using the WGCNA method and finally determined 8 modules. Among them, the turquoise module (MEturquoise) was most positively correlated with the cardiotoxicity abnormal phenotype, containing 857 genes, while the blue module (MEblue) was most negatively correlated, containing 778 genes. Besides, we performed GO and KEGG analyses, respectively, to fully evaluate the biological roles of the two modules. The biological annotation of GO item demonstrated that those genes in MEturquoise were related to “cytoplasm,” “nucleotide binding,” “cellular response to insulin stimulus,” etc., and genes in MEblue were related to “extracellular region,” “heparin-binding,” and “cell adhesion.” KEGG analysis indicated several biological signaling pathways were significantly enriched in MEturquoise, such as the “FoxO signaling pathway,” which has been validated by a previous study [36]. However, in MEblue, other types of signaling, such as “focal adhesion,” “ECM receptor interaction,” and “PI3K-Akt signaling pathway,” were significantly enriched, and several of them have also been identified involvement in cardiotoxicity [37, 38]. Therefore, these pathways especially those not previously reported, greatly enrich the knowledge regarding the disease and provide potential intervention targets.

The better the correlation between modules and disease traits, the more important function genes have. On these grounds, 35 genes from MEturquoise and 42 genes from MEblue were screened out. The significantly upregulated DEGs in 35 genes from MEturquoise and the significantly downregulated DEGs in 42 genes from MEblue were considered the key ones, and finally, 14 genes were enrolled. To validate the role of these 14 key genes, we first merged and reanalyzed two datasets GSE81448 and GSE97642, which were modeled with the same dose of doxorubicin, but the detection time was advanced to 20 hours after the operation. Limd1, Spock2, Sult1a1, etc. were also discovered to be dysregulated, the same as the previous results. To identify the specific cell populations that lead to changes in the expression of these genes, we reanalyzed another three datasets that detected the CMs, FBs, and SMCs under the intervention of doxorubicin. As the results revealed, dysregulation of several genes including Limd1 and Sult1a1 was not from the above three kinds of cells, and potentially resulted from the inflammatory cells infiltrating into tissue. Therefore, we further evaluate their expression in the PBMC of the rat cardiotoxicity model. In the end, only Limd1 was significantly upregulated, the same as in the heart of the mouse model. Besides, ROC analysis indicated that Limd1 in PBMC had the ability to diagnose this cardiotoxic disease. As is well known, Limd1 (LIM domain-containing protein 1) is a member of the Zyxin proteins and is widely expressed in human tissues. Several previous studies have suggested the suppressor role of Limd1 in tumor diseases [39]. In cardiovascular disease, Limd1 has been reported to be functionally closely related to cardiac fibroblasts [40]. Based on the results, Limd1 was deemed as the hub gene and a valuable biomarker.

Due to that Limd1 was closely related to inflammatory cells in our study, we subsequently determined the biological function of Limd1 by the GSEA tool and protein interactive network. GSEA results of the mouse model suggested that Limd1 may be functionally involved in immunocyte chemotaxis, adhesion, and migration. In the network built by the GeneMANIA database, the Limd1 was depicted to be connected with the NF-kappaB signaling pathway through interacting with Limd1, EGFR, and TRAF6 proteins. As one of the best-understood immune-related pathways, the NF-kappaB signaling pathway is activated by numerous discrete stimuli and participates in regulating activities of the majority of immunocytes including macrophage, dendritic cells, and neutrophils [41], thus exerting a comprehensive role in inflammation.

In fact, the cardiotoxicity induced by doxorubicin is in general characterized by an abnormal inflammatory response, and the NF-kappaB signaling pathway was indeed the mediator [42]. Due to that Limd1 was a highly likely regulator of immune cell infiltrates in the toxic heart according to the above analysis, we therefore finally assessed the immune infiltration in the hearts of mouse model on the 4th day after doxorubicin injection using the CIBERSORTx method. Compared with the hearts in the control group, the doxorubicin-intervened hearts have a significantly higher proportion of “dendritic cells activated” and a lower proportion of “macrophage M1”. Besides, the proportion of monocytes seems to be decreased in the cardiotoxicity model heart, despite not being statistically significant. As a widely studied inflammatory cell type, the proinflammatory M1 macrophage has been found to be increased in doxorubicin-induced cardiomyopathy [43]. The contradiction was due to that the datasets we reanalyzed used a one-time high-dose modeling method, and the literature used intermittent multiple-dosing ways. The one-time modeling indicates that the damage reaches the peak shortly after the beginning, and injury stimulation cannot be maintained at the following. This damage mode was similar to that in other heart diseases, such as acute myocardial infarction (AMI). During the progression of AMI, the response of the monocyte/macrophage system is characterized by the accumulation of proinflammatory monocytes and macrophages over 48 to 72 h, followed by a reparative phase at 4 to 7 days driven by anti-inflammatory macrophages [44]. Despite the M1 macrophage (pro-) was rapidly accumulated and peaking on the fifth day at the damaged site, the ratio of M1 type to the total macrophages (M1 and M2) had decreased significantly earlier than the fifth day [45]. Therefore, our results that the proportion of monocyte/macrophage M1 decreased could be clued from the pattern of myocardial infarction.

Dendritic cells are a kind of bone marrow-derived cells arising from lympho-myeloid hematopoiesis that are responsible for initiating and controlling immune responses, specializing in antigen presentation to drive T-cell priming and differentiation [46]. In research decades ago, the number of interstitial dendritic cells/mm^2^ of a left ventricle section was found to be remarkably increased in animals receiving doxorubicin, compared with saline-treated control [47], which was similar to our analysis. As a matter of fact, dendritic cells are widely involved in the progression of heart diseases. In the mouse model of transverse aortic constriction (TAC)-induced pressure overload, the relative abundance of dendritic cells was found to be increased in the hypertrophic myocardium both at 1 and at 4 weeks post-TAC [48]. Besides, dendritic cells worked as a pathogen to induce autoimmune heart failure under certain conditions [49]. In an acute myocardial infarction mouse model, subcutaneously administration of tolerogenic dendritic cells (tDCs) could reduce infarct size, improve heart function and mouse survival, by timely promoting Treg cell, and activating its mediated macrophage conversion to the reparative M2 to replace the inflammation M1 type [45]. Interestingly, the fraction of activated dendritic cells was positively correlated with that of Tregs and M2 macrophage and negatively correlated with M1 macrophage, as revealed in our results. These suggest a potential pattern that the increasing of dendritic cells may exert its influence on the cardiotoxicity through the subsequent Treg cell and macrophage. Given the biological role of the NF-kappaB signaling pathway in monocyte, macrophage, and dendritic cells, we, therefore, wonder whether Limd1, a potential immunocyte regulator, is associated with the infiltration of these inflammatory cells. Finally, we found that Limd1 expression was negatively correlated with the fraction of monocyte and macrophage and positively correlated with the dendritic cells fraction. Thus, we speculate that Limd1 acts as the regulator of the inflammation system through the NF-kappaB pathway, playing a role in doxorubicin-induced cardiotoxicity.

There are still some limitations to this study. Firstly, the differential expression analyses compared the treatment (*n* = 2) and control (*n* = 2) groups in the GSE154101 and GSE154118 datasets could lead to some inaccuracy due to the limitations of the sample size. Secondly, the number of sample used for the WGCNA (*n* = 10) could also lead to some bias due to the small sample size (because *n* ≥ 15 samples is suggested for the WGCNA). Thirdly, the above results were not further validated in our own disease model.

## 5. Conclusions

In conclusion, Limd1 was upregulated in PBMC and the heart of the doxorubicin-induced cardiotoxicity model and is a valuable circulating biomarker. In particular, we found that Limd1 was functionally correlated with the immune system and significantly correlated with the infiltration of immunocytes including monocytes, M1 macrophage, and dendritic cell. It is worth further exploring to clarify its role in this cardiotoxicity.

## Figures and Tables

**Table 1:** Summary of Preliminary Results.

Criteria	Number	Upregulated	Downregulated
Total number of genes	20813		
|log_2_ (FC)| >1 & *p*.*adj* < 0.05	120	54	66
|log_2_ (FC)| >1.5 & *p*.adj < 0.05	28	13	15
|log_2_ (FC)| >2 & *p*.adj < 0.05	9	4	5

**Table 2:** Potential therapeutic drugs.

Score	Name	Description
-96.74	PF-04217903	c-met inhibitor
-96.2	Propranolol	Adrenergic receptor antagonist
-94.47	Azithromycin	Bacterial 50S ribosomal subunit inhibitor
-94.17	9-methyl-5H-6-thia-4,5-diaza-chrysene-6,6-dioxide	NFkB-pathway inhibitor
-93.9	Mafenide	Carbonic anhydrase inhibitor
-93.48	Prunetin	Breast cancer resistance protein inhibitor
-92.4	TAK-715	p38 MAPK inhibitor
-92.24	Dibenzepin	Norepinephrine reuptake inhibitor
-92	Benzylpenicillin	Penicillin-binding protein inhibitor
-91.69	FTI-276	Farnesyltransferase inhibitor

**Table 3:** Analysis of the 14 hub genes in doxorubicin-induced CMs, fibroblasts, and SMCs.

Gene	Cardiomyocytes		Fibroblasts		Smooth muscle cells	
	Log FC	Adj. *p*	Log FC	Adj. *p*	Log FC	Adj. *p*
Limd1	-0.69	0.0024	-0.17	0.872	0.27	0.372
Aox1	0.18	0.265	0.13	0.869	2.48	4.76E-06
Spock2	0.49	0.297	—	—	—	—
Sult1a1	0.3	0.182	0.18	0.922	-1.34	0.063
Ephx1	0.26	0.134	0.7	0.606	1.57	6.04E-10
Pcolce	0.14	0.217	0.05	0.962	0.23	0.362
Evi2a	0.41	0.186	1.45	0.214	-0.09	0.93
Klhdc8a	-0.09	0.591	0.65	0.687	0.45	0.639
Slamf9	0.24	0.536	0.65	0.702	—	—
Col15a1	—	—	0.41	0.805	-0.35	0.527
Cd68	0.047	0.86	—	—	-0.05	0.871
Col6a2	—	—	-0.02	0.979	0.31	0.475
Nrep	0.074	0.755	-1.12	0.308	-2.77	1.10E-06
Mgl2	—	—	—	—	—	—

-represents no result.

## Data Availability

The data used to support the findings of this study are available from the first author upon reasonable request.

## Supplementary Materials

Supplementary Materials Supplementary Table 1. The detailed information of 120 DEGs. Supplementary Figure 1 Remove batch between GSE81448 and GSE9764. Supplementary Figure 2 Volcano plots displayed the upregulated and downregulated genes in doxorubicin-treated CMs, Fibroblasts, and SMCs.
