# Supplementary material for: Upregulation of Biomarker Limd1 Was Correlated with Immune Infiltration in Doxorubicin-Related Cardiotoxicity
Source: Mediators Inflamm. 2023 Mar 23;2023:8347759. doi: 10.1155/2023/8347759 (PMC10063360; doi:10.1155/2023/8347759)
Supplement: Supplementary Materials — Supplementary Table 1. The detailed information of 120 DEGs. Supplementary Figure 1 Remove batch between GSE81448 and GSE9764. Supplementary Figure 2 Volcano plots displayed the upregulated and downregulated genes in doxorubicin-treated CMs, Fibroblasts, and SMCs. [file 8347759.f1.docx]

Supplementary Table 1. The detailed information of 120 DEGs

| id | logFC | AveExpr | t | P.Value | adj.P.Val | B | Gene.Symbol |
| --- | --- | --- | --- | --- | --- | --- | --- |
| 1451675_a_at | -3.96036 | 6.872194 | -10.216 | 1.04E-07 | 4.91E-05 | 8.209751 | Alas2 |
| 1448021_at | -3.05813 | 5.869672 | -7.35212 | 4.58E-06 | 0.000446 | 4.521172 | Fam46c |
| 1438651_a_at | -2.71458 | 7.257754 | -11.0044 | 4.22E-08 | 3.52E-05 | 9.06118 | Aplnr |
| 1448553_at | 2.64421 | 12.54627 | 5.059897 | 0.000197 | 0.004812 | 0.76428 | Myh7 |
| 1427883_a_at | -2.33415 | 9.675181 | -10.4265 | 8.12E-08 | 4.57E-05 | 8.443216 | Col3a1 |
| 1434202_a_at | 2.193499 | 8.335248 | 11.78149 | 1.83E-08 | 2.12E-05 | 9.841832 | Fam107a |
| 1424638_at | 2.090497 | 9.360201 | 6.211237 | 2.72E-05 | 0.0013 | 2.746498 | Cdkn1a |
| 1428942_at | 2.021062 | 10.04206 | 9.110727 | 4.03E-07 | 0.000102 | 6.904838 | Mt2 |
| 1418493_a_at | -2.0174 | 6.933459 | -4.49863 | 0.000552 | 0.009623 | -0.26556 | Snca |
| 1423858_a_at | 1.993479 | 7.713537 | 7.394112 | 4.30E-06 | 0.000436 | 4.582918 | Hmgcs2 |
| 1425394_at | -1.98909 | 6.394599 | -4.90526 | 0.000261 | 0.005832 | 0.484262 | BC023105 |
| 1427865_at | -1.96677 | 6.187678 | -8.17701 | 1.40E-06 | 0.000212 | 5.689632 | Hbb-b2 |
| 1452661_at | -1.9043 | 8.884765 | -7.53197 | 3.51E-06 | 0.000393 | 4.783889 | Tfrc |
| 1435026_at | 1.848228 | 7.147692 | 11.56969 | 2.29E-08 | 2.27E-05 | 9.634499 | Spock2 |
| 1418652_at | -1.82341 | 6.421067 | -8.82907 | 5.81E-07 | 0.000125 | 6.549704 | Cxcl9 |
| 1435290_x_at | -1.79288 | 9.447771 | -11.0579 | 3.98E-08 | 3.45E-05 | 9.116663 | H2-Aa |
| 1416125_at | 1.780864 | 8.939818 | 10.17015 | 1.10E-07 | 5.07E-05 | 8.158337 | Fkbp5 |
| 1416371_at | 1.73118 | 9.003315 | 9.120306 | 3.98E-07 | 0.000102 | 6.916748 | Apod |
| 1416953_at | 1.716878 | 10.62956 | 8.788727 | 6.13E-07 | 0.000129 | 6.498045 | Ctgf |
| 1450648_s_at | -1.66345 | 8.356248 | -10.315 | 9.25E-08 | 4.69E-05 | 8.320228 | H2-Ab1 |
| 1438467_at | -1.60848 | 6.206442 | -9.98007 | 1.37E-07 | 5.20E-05 | 7.942602 | Mgl2 |
| 1439163_at | 1.59654 | 9.343608 | 5.62363 | 7.31E-05 | 0.002461 | 1.758296 | Zbtb16 |
| 1427345_a_at | 1.585797 | 10.54722 | 11.15917 | 3.56E-08 | 3.22E-05 | 9.221074 | Sult1a1 |
| 1455399_at | 1.582874 | 7.161193 | 9.05037 | 4.36E-07 | 0.000103 | 6.829541 | Cnksr1 |
| 1425519_a_at | -1.53717 | 8.821481 | -8.47783 | 9.28E-07 | 0.000165 | 6.093143 | Cd74 |
| 1423606_at | -1.53178 | 7.835016 | -8.64751 | 7.39E-07 | 0.000142 | 6.315631 | Postn |
| 1423085_at | -1.52179 | 8.535702 | -7.49292 | 3.72E-06 | 0.000405 | 4.72723 | Efnb3 |
| 1427747_a_at | 1.51806 | 7.193206 | 5.717973 | 6.22E-05 | 0.002243 | 1.920327 | Lcn2 |
| 1419100_at | 1.486992 | 8.035261 | 4.619 | 0.000441 | 0.008319 | -0.0417 | Serpina3n |
| 1417025_at | -1.4669 | 8.401286 | -7.00631 | 7.72E-06 | 0.000589 | 4.003165 | H2-Eb1 |
| 1437422_at | 1.462529 | 6.668526 | 6.093107 | 3.31E-05 | 0.001465 | 2.551878 | Sema5a |
| 1450839_at | -1.45931 | 7.678119 | -13.258 | 4.21E-09 | 2.12E-05 | 11.18299 | Nrep |
| 1448755_at | -1.4581 | 7.095835 | -16.4225 | 2.77E-10 | 5.77E-06 | 13.54319 | Col15a1 |
| 1417141_at | -1.45157 | 9.082337 | -4.95329 | 0.000239 | 0.005474 | 0.571561 | Igtp |
| 1424131_at | -1.44006 | 7.493589 | -7.14134 | 6.28E-06 | 0.00053 | 4.207462 | Col6a3 |
| 1449183_at | 1.436318 | 11.28475 | 11.99578 | 1.46E-08 | 2.12E-05 | 10.0476 | Comt |
| 1449065_at | 1.385836 | 7.318096 | 6.001524 | 3.85E-05 | 0.001609 | 2.399585 | Acot1 |
| 1422217_a_at | -1.37919 | 6.177845 | -5.09395 | 0.000186 | 0.004579 | 0.825542 | Cyp1a1 |
| 1418595_at | 1.378189 | 10.17095 | 9.220301 | 3.51E-07 | 9.73E-05 | 7.040426 | Plin4 |
| 1416464_at | -1.36792 | 5.436802 | -3.54793 | 0.003404 | 0.0321 | -2.07228 | Slc4a1 |
| 1417765_a_at | 1.366546 | 6.344353 | 5.522748 | 8.70E-05 | 0.002724 | 1.583634 | Amy1 |
| 1416926_at | 1.363178 | 9.257539 | 5.705125 | 6.36E-05 | 0.002266 | 1.898336 | Trp53inp1 |
| 1419435_at | 1.345141 | 9.043735 | 11.90534 | 1.61E-08 | 2.12E-05 | 9.961235 | Aox1 |
| 1415983_at | -1.34034 | 7.682212 | -7.35406 | 4.57E-06 | 0.000446 | 4.524026 | Lcp1 |
| 1419149_at | 1.331051 | 7.91925 | 5.698248 | 6.43E-05 | 0.002278 | 1.886555 | Serpine1 |
| 1419315_at | -1.32977 | 6.678198 | -11.6964 | 2.00E-08 | 2.19E-05 | 9.75904 | Slamf9 |
| 1449901_a_at | 1.313836 | 7.231047 | 7.940661 | 1.95E-06 | 0.000264 | 5.364279 | Map3k6 |
| 1425233_at | -1.31064 | 6.212641 | -3.94598 | 0.001575 | 0.019164 | -1.30988 | 2210407C18Rik |
| 1441111_at | -1.30945 | 9.912448 | -6.21391 | 2.71E-05 | 0.001299 | 2.750883 | Mylk4 |
| 1417793_at | -1.3032 | 8.189229 | -4.31818 | 0.000775 | 0.011951 | -0.60385 | Irgm2 |
| 1436033_at | 1.302258 | 8.316897 | 6.483058 | 1.75E-05 | 0.000994 | 3.18654 | Fam214a |
| 1418697_at | 1.293629 | 9.660041 | 7.891422 | 2.09E-06 | 0.000278 | 5.295558 | Inmt |
| 1435459_at | 1.293186 | 9.487866 | 9.135689 | 3.91E-07 | 0.000102 | 6.935851 | Fmo2 |
| 1423669_at | -1.29177 | 6.879465 | -7.43722 | 4.04E-06 | 0.000423 | 4.646054 | Col1a1 |
| 1419684_at | -1.29129 | 7.540184 | -4.90431 | 0.000261 | 0.005836 | 0.48252 | Ccl8 |
| 1452250_a_at | -1.28447 | 8.871628 | -11.7983 | 1.80E-08 | 2.12E-05 | 9.858161 | Col6a2 |
| 1433652_at | -1.28128 | 6.381154 | -6.64346 | 1.36E-05 | 0.000843 | 3.441125 | Igsf1 |
| 1455377_at | 1.276692 | 5.975136 | 8.581177 | 8.08E-07 | 0.000147 | 6.229084 | Ttll7 |
| 1422470_at | 1.267799 | 12.36973 | 11.64325 | 2.12E-08 | 2.20E-05 | 9.706958 | Bnip3 |
| 1424885_at | -1.25019 | 6.664851 | -10.2421 | 1.01E-07 | 4.87E-05 | 8.239037 | Klhdc8a |
| 1453238_s_at | 1.240534 | 11.19118 | 4.968117 | 0.000233 | 0.005401 | 0.598443 | 3930401B19Rik |
| 1423607_at | -1.24024 | 9.962274 | -7.67867 | 2.84E-06 | 0.000345 | 4.994852 | Lum |
| 1417814_at | -1.23316 | 6.746723 | -6.69203 | 1.26E-05 | 0.000808 | 3.517467 | Pla2g5 |
| 1428083_at | 1.225194 | 10.80486 | 6.000734 | 3.86E-05 | 0.001609 | 2.398266 | Neat1 |
| 1416514_a_at | -1.22383 | 8.45256 | -10.3405 | 8.98E-08 | 4.67E-05 | 8.348487 | Fscn1 |
| 1429900_at | 1.222264 | 6.467899 | 6.330151 | 2.24E-05 | 0.001156 | 2.94034 | 5330406M23Rik |
| 1448949_at | 1.219642 | 8.999239 | 10.6259 | 6.46E-08 | 4.48E-05 | 8.660208 | Car4 |
| 1418392_a_at | -1.20424 | 7.944105 | -5.92634 | 4.37E-05 | 0.001752 | 2.273649 | Gbp3 |
| 1427161_at | -1.20361 | 7.372729 | -5.58531 | 7.81E-05 | 0.002581 | 1.692124 | Cenpf |
| 1416246_a_at | -1.17964 | 6.440707 | -6.48771 | 1.74E-05 | 0.000994 | 3.193976 | Coro1a |
| 1448842_at | 1.165811 | 5.448652 | 5.202117 | 0.000153 | 0.004044 | 1.019111 | Cdo1 |
| 1428776_at | 1.161826 | 8.09177 | 6.332723 | 2.23E-05 | 0.001156 | 2.94451 | Slc10a6 |
| 1449164_at | -1.16032 | 6.55117 | -12.0285 | 1.42E-08 | 2.12E-05 | 10.07864 | Cd68 |
| 1422983_at | -1.1536 | 5.229162 | -6.76718 | 1.12E-05 | 0.000738 | 3.634917 | Itgb6 |
| 1437165_a_at | -1.15038 | 8.716639 | -10.8368 | 5.09E-08 | 3.96E-05 | 8.885382 | Pcolce |
| 1418509_at | -1.13957 | 8.094158 | -6.98566 | 7.97E-06 | 0.000594 | 3.971691 | Cbr2 |
| 1418204_s_at | -1.13895 | 6.02595 | -7.33892 | 4.67E-06 | 0.000446 | 4.501708 | Aif1 |
| 1417172_at | -1.13862 | 4.942038 | -7.20099 | 5.74E-06 | 0.000504 | 4.296886 | Ube2l6 |
| 1427301_at | -1.1134 | 5.906089 | -8.98068 | 4.77E-07 | 0.00011 | 6.742052 | Cd48 |
| 1428306_at | 1.107417 | 10.47859 | 6.873102 | 9.48E-06 | 0.000669 | 3.799051 | Ddit4 |
| 1425400_a_at | -1.10476 | 6.957911 | -6.98438 | 7.98E-06 | 0.000594 | 3.969735 | Cited4 |
| 1418155_at | 1.103254 | 9.461851 | 4.172391 | 0.001021 | 0.014334 | -0.87923 | Myot |
| 1424609_a_at | 1.103039 | 8.770058 | 6.688366 | 1.26E-05 | 0.00081 | 3.511717 | Gm4354 |
| 1418979_at | -1.10247 | 5.724633 | -7.073 | 6.97E-06 | 0.000557 | 4.104394 | Akr1c14 |
| 1418945_at | 1.102033 | 6.699318 | 4.742245 | 0.000351 | 0.0072 | 0.185901 | Mmp3 |
| 1450241_a_at | -1.10025 | 5.927085 | -9.3607 | 2.94E-07 | 8.61E-05 | 7.212098 | Evi2a |
| 1448259_at | -1.09609 | 8.913025 | -9.46783 | 2.57E-07 | 7.99E-05 | 7.34154 | Fstl1 |
| 1424375_s_at | -1.09279 | 9.033289 | -12.1613 | 1.24E-08 | 2.12E-05 | 10.20384 | Gimap4 |
| 1450843_a_at | -1.08911 | 9.835812 | -8.6847 | 7.03E-07 | 0.000141 | 6.363912 | Serpinh1 |
| 1448891_at | -1.0876 | 6.180928 | -8.00255 | 1.79E-06 | 0.000247 | 5.450188 | Fcrls |
| 1424727_at | -1.08598 | 5.705737 | -6.54181 | 1.60E-05 | 0.000941 | 3.280229 | Ccr5 |
| 1422731_at | 1.082549 | 9.670539 | 10.11529 | 1.17E-07 | 5.18E-05 | 8.096469 | Limd1 |
| 1417852_x_at | 1.081057 | 5.100887 | 4.557521 | 0.000495 | 0.008916 | -0.15585 | Clca1 |
| 1417273_at | 1.070861 | 12.27924 | 5.696019 | 6.46E-05 | 0.002278 | 1.882736 | Pdk4 |
| 1419282_at | -1.0705 | 6.59971 | -4.7029 | 0.000378 | 0.00757 | 0.113431 | Ccl12 |
| 1435261_at | 1.064272 | 9.928586 | 12.09551 | 1.32E-08 | 2.12E-05 | 10.14202 | Tmtc1 |
| 1448272_at | 1.061452 | 8.540961 | 6.614866 | 1.42E-05 | 0.000868 | 3.396015 | Btg2 |
| 1422438_at | 1.060994 | 9.375487 | 10.44308 | 7.97E-08 | 4.57E-05 | 8.461454 | Ephx1 |
| 1422557_s_at | 1.058902 | 12.62452 | 9.304985 | 3.15E-07 | 9.03E-05 | 7.144245 | Mt1 |
| 1431255_at | 1.055013 | 8.009898 | 12.21835 | 1.17E-08 | 2.12E-05 | 10.25716 | Calr3 |
| 1420888_at | 1.05174 | 9.160028 | 7.705224 | 2.73E-06 | 0.000338 | 5.032722 | Bcl2l1 |
| 1436530_at | -1.05037 | 8.540459 | -3.92476 | 0.001641 | 0.019703 | -1.35039 | Wfdc17 |
| 1428259_at | -1.0487 | 7.896653 | -9.09336 | 4.12E-07 | 0.000102 | 6.883219 | Pxdn |
| 1418450_at | -1.04808 | 8.054698 | -6.5603 | 1.55E-05 | 0.000924 | 3.309607 | Islr |
| 1434817_s_at | 1.046131 | 7.10325 | 6.392814 | 2.03E-05 | 0.00109 | 3.041651 | Rprd2 |
| 1449106_at | 1.03671 | 11.5228 | 7.63216 | 3.04E-06 | 0.000359 | 4.928293 | Gpx3 |
| 1420498_a_at | -1.03621 | 8.203616 | -8.6567 | 7.30E-07 | 0.000142 | 6.32758 | Dab2 |
| 1430530_s_at | 1.036091 | 6.552852 | 6.13068 | 3.11E-05 | 0.00141 | 2.614001 | Nmral1 |
| 1458455_at | 1.033516 | 8.15091 | 5.837052 | 5.08E-05 | 0.001958 | 2.123014 | Abra |
| 1451245_at | -1.03211 | 7.99138 | -8.45671 | 9.55E-07 | 0.000168 | 6.065189 | Lrrc3b |
| 1436240_at | 1.02683 | 6.292456 | 4.81984 | 0.000305 | 0.006527 | 0.328311 | B230214O09Rik |
| 1423547_at | -1.0258 | 10.49846 | -10.5066 | 7.41E-08 | 4.57E-05 | 8.530883 | Lyz2 |
| 1449363_at | 1.022582 | 6.622007 | 5.944979 | 4.24E-05 | 0.001712 | 2.304945 | Atf3 |
| 1429159_at | -1.01997 | 6.706598 | -6.7842 | 1.09E-05 | 0.000724 | 3.661394 | Itih5 |
| 1452865_at | -1.01966 | 7.366621 | -5.4833 | 9.32E-05 | 0.002858 | 1.514938 | Lrrc27 |
| 1424715_at | 1.017794 | 9.091446 | 6.711471 | 1.22E-05 | 0.000788 | 3.547926 | Retsat |
| 1436996_x_at | -1.00649 | 11.61466 | -10.7895 | 5.37E-08 | 3.99E-05 | 8.83524 | Lyz1 |
| 1422903_at | -1.00643 | 7.452917 | -9.51382 | 2.43E-07 | 7.99E-05 | 7.396705 | Ly86 |
| 1417750_a_at | -1.00637 | 7.707289 | -5.21942 | 0.000148 | 0.003947 | 1.049926 | Slc25a37 |
| 1419569_a_at | -1.00195 | 6.385159 | -7.18267 | 5.90E-06 | 0.00051 | 4.269477 | Isg20 |


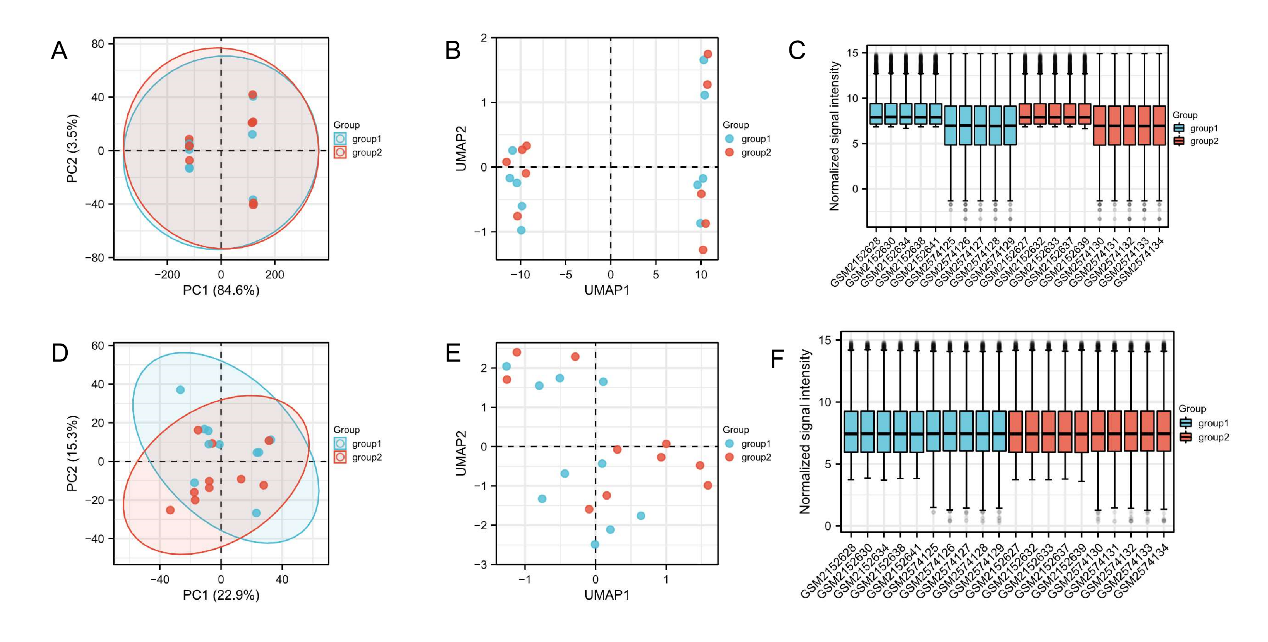


Supplementary Figure 1. Remove batch between GSE81448 and GSE97642. (A)(B)(C) PCA diagram, UMAP diagram and boxplot diagram of the data distribution before removing batch. (D)(E)(F) PCA diagram, UMAP diagram and boxplot diagram of the data distribution after removing batch. Group1 represents control group while group2 represents doxorubicin treated group.


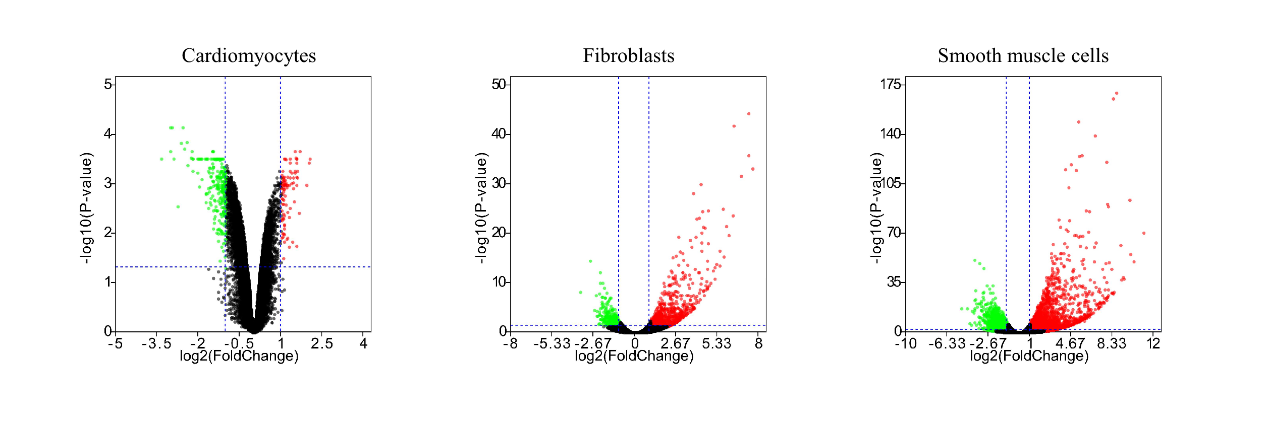


Supplementary Figure 2. Volcano plots displayed the upregulated and downregulated genes in doxorubicin treated CMs, Fibroblasts and SMCs. Red plots represented upregulated genes and green plots represented downregulated genes.
